# Treatment of Capillary Nævus

**Published:** 1876-09

**Authors:** 


Treatment of Capillary Nævus.—Mr. Bradley states, in the *British Medical Journal*, that he was induced to try the effect of treating the ordinary capillary nævus, or mother's mark, by tattooing with carbolic acid. In the only case in which he has tried it the port wine stains disappeared in about three weeks. He recommends it for further trial.

